# A Combined Approach of Double Network Hydrogel and Nanocomposites Based on Hyaluronic Acid and Poly(ethylene glycol) Diacrylate Blend

**DOI:** 10.3390/ma11122454

**Published:** 2018-12-04

**Authors:** Alfredo Ronca, Ugo D’Amora, Maria Grazia Raucci, Hai Lin, Yujiang Fan, Xingdong Zhang, Luigi Ambrosio

**Affiliations:** 1 Institute of Polymers, Composites and Biomaterials, National Research Council, 80125 Naples, Italy; mariagrazia.raucci@cnr.it (M.G.R.); luigi.ambrosio@cnr.it (L.A.); 2 National Engineering Research Center for Biomaterials, Sichuan University, Chengdu 61000, China; ohai3@163.com (H.L.); yujiang.fan@163.com (Y.F.); zhangxd@scu.edu.cn (X.Z.)

**Keywords:** double network, hyaluronic acid derivative, nanocomposite, sol-gel method, PEGDA

## Abstract

In this study, an innovative polymer blend, based on double network (DN) approach, has been developed by integrating a poly(ethylene glycol) diacrylate (PEGDA) network into a chemically modified hyaluronic acid sodium salt (HAs) hydrogel matrix. Here, the HAs was chemically functionalized with photocrosslinkable moieties by reacting with maleic anhydride (MAA) to obtain a maleated hyaluronic acid (MaHA). Furthermore, nanocomposite DN hydrogels were suitably prepared by physical blending of hydroxyapatite nanoparticles (HAp), obtained by sol-gel synthesis, within the hydrogel. Physico-chemical, thermal, morphological and mechanical analyses were performed. Results showed enhanced mechanical properties and a homogenous microstructure as highlighted by mechanical and morphological investigations. This suggests that nanocomposite DN hydrogels are promising candidates for biomedical applications.

## 1. Introduction

An ideal biomaterial has to satisfy specific requirements such as mechanical properties tuned to the specific application, high biocompatibility and sufficient stability against physiological media, preventing material degradation without inducing adverse phenomena at the site of implant [1,2,3,4]. Natural polymers are largely available in nature and by physical or chemical crosslinking it is possible to prepare a group of polymers called hydrogels [5,6,7]. Hydrogels have gained great attention in the biomedical field, as excellent soft and wet materials due to their ability to absorb water from 10–20% up to thousands of times their dry weight, which confers a structural similarity to the natural extracellular matrix [8,9,10,11]. They possess interesting features such as swelling/deswelling, stimuli-responsiveness, shock absorption, and low sliding friction [6,10,12,13]. However conventional hydrogels, usually composed of a single network (SN), are mechanically too weak for practical uses [1,14]. For this reason, the use of these materials has been limited to specific fields of interest, such as drug delivery and water absorption, where their mechanical properties are not strongly required. Blending two polymers has been a common approach to develop a new class of biomaterials showing combinations of properties not achievable by using individual polymers [3,15]. In particular, blends of synthetic and natural polymers allow to obtain materials that merge a wide range of physico-chemical properties of synthetic polymers as well as the biocompatibility, the safety and side effects avoidance of natural ones [16,17,18,19]. Furthermore, these materials are mainly characterized by the presence of functional groups leading to the implementation of different modifications. In this scenario, interpenetrated polymer network (IPN) or semi-IPN hydrogels have been useful methods to prepare materials with increased load-bearing properties [20,21]. Basically, IPN hydrogels can be classified into three different categories: (a) IPNs, formed by two interpenetrated networks, with many entanglements and physical interactions between them [22]; (b) homo-IPNs, characterized by two polymers with the same structure, forming independent networks [23]; (c) semi-IPNs, consisting of one component with a linear structure instead of a network [21,24].

“Double network” (DN) hydrogels, developed for the first time by Gong et al., represent a specific class of IPNs [25,26] as they combine a brittle polyelectrolyte as first network and a neutral polymer as second one [3,13,14,25,26,27]. They are synthesized by a two-step sequential network formation in which first a tightly crosslinked network gel of polyelectrolyte is formed. The first gel is then dipped in an aqueous solution of a second neutral polymer allowing a second network formation in the first one [25,26,27]. Traditional IPNs are only linear combination of two component networks while in DN hydrogel the second network is densely packed and interpenetrated within the cage of the first network, resulting in an extremely tough structure [14,20,26]. As example, the DN hydrogels characterized by the presence of poly(2-acrylamido-2-methyl-1-propanesulfonic acid) as polyelectrolyte and polyacrylamide neutral polymer (containing up to 90 wt% of H_2_O), showed an elastic modulus of 0.1–1 MPa and a compressive strength of about 20 MPa [28]. Similarly, polyethyleneoxide/polyacrylic acid double network gels as well as the modified hyaluronan/poly(*N*,*N*’-dimethylacrylamide) developed by Weng et al., and bacterial cellulose/gelatin DN hydrogels designed by Nakayama et al. showed enhanced mechanical performances when compared to individual components [27,28].

Moreover, it is widely recognized that the incorporation of inorganic nanoparticles (i.e., hydroxyapatite nanoparticles, HAp) within the polymer matrix can effectively enhance mechanical properties, showing flexibility and structural integrity better than single component [29,30,31]. The main driving idea of this work was the synthesis of an advanced blend with high mechanical properties by combining nanocomposite and double network approaches. Photoactive polymerizable motifs as maleate groups were grafted onto a hyaluronic acid sodium salt (HAs) in order to obtain photocrosslinkable neat and nanocomposite maleated hyaluronic acid (MaHA) to prepare the first network (MaHA and MaHA/HAp-SN). The designed DN blend structure was formed by a two-step photocrosslinking of MaHA-rich first network and a poly(ethylene glycol) diacrylate (PEGDA)-rich second component. The mechanical properties of neat and nanocomposite DN hydrogels were compared with those of MaHA-SN hydrogels. The influence of the PEGDA second system as well as the HAp amount on the mechanical and thermal properties of DN hydrogels were studied. Moreover, the dispersion of HAp inside the matrix and the morphological difference between dry SN and DN structures were evaluated.

## 2. Materials and Methods

### 2.1. Synthesis of MaHA and Characterization 

**Scheme 1:**
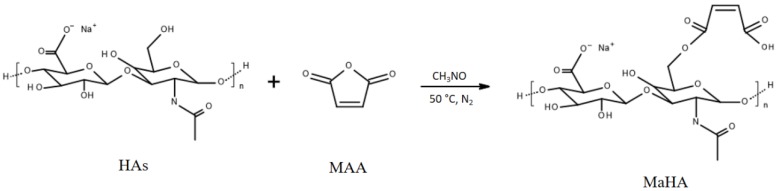
General reaction scheme of the hyaluronic acid sodium salt (HAs) with maleic anhydride (MAA) to obtain maleated hyaluronic acid (MaHA).

HAs (M_w_ = ~340 kDa) was provided by Bloomage Freda Biopharm Co. Ltd., Jinan, China. MaHA was obtained by reaction of the primary –OH groups with maleic anhydride (MAA, Kelong Chemical CO. Ltd., Liaoning, China), through a ring opening mechanism in dry formamide (CH_3_NO, Sigma Aldrich, Saint Louis, MO, USA) under nitrogen (N_2_) atmosphere for 24 h at 50 °C as reported in Scheme 1. Briefly, HAs was dissolved in 100 mL of CH_3_NO at a concentration of 1% (wt/v) at 50 °C under N_2_ atmosphere, and stirred until fully dissolved. Separately, MAA was dissolved in 50 mL of CH_3_NO and it was added drop by drop to HAs. Different MAA/HAs molar ratios of 10/1, 15/1 and 20/1 have been used in order to tune the degree of substitution (DS) of modified polymers (Table 1). The solution was precipitated and washed several times in cold anhydrous ethyl alcohol. The product was dissolved in distilled water (dH_2_O), dialyzed for 4 days and lyophilized by freeze drying (VirTis, Los Angeles, CA, USA).

^1^H Nuclear magnetic resonance (NMR, Bruker AVIII 400HD, Fällanden, Swiss) was employed to calculate the DS of the maleated HAs. By using a shaking incubator (7000 rpm for 20 min), the MaHA (5–6 mg/mL) was completely dissolved in deuterium oxide (D_2_O). After that, it was transferred into NMR tubes. The data were collected at a frequency of 400 MHz. Phase and baseline corrections were applied before obtaining the areas (integrals) of purely absorptive peaks.

The DS of MaHA was determined by comparing the integral area of protons of unsaturated bonds (maleated moieties) with methyl (–CH_3_) peak. MaHA 20 was selected for the network and composite preparation due to its higher DS.

### 2.2. Preparation of Neat and Composite Single Network (SN) Hydrogels

To obtain the networks, MaHA 20 was dissolved in dH_2_O (20 mg/mL) containing the biocompatible photoinitiator Irgacure 2959 (Sigma Aldrich, Saint Louis, MO, USA) at a concentration of 0.1% wt/v. Then 150 μL of solution was transferred into a Teflon mold between two quartz plates, and it was irradiated by 365 nm UV light for 60 s (OmniCure S1500, Waltham, MA, USA, light intensity 16 mW/cm^2^).

SN disc-shaped samples measuring 8.0 ± 0.8 mm × 4.0 ± 0.5 mm were then rinsed in dH_2_O for two days to clear away any unreacted compound and stored at 4 °C. To prepare composite structures, HAp were produced at room temperature by sol-gel synthesis using as precursors calcium nitrate tetrahydrate (Ca(NO_3_)_2_×4H_2_O—in Shan Hua Shi—China), di-ammonium hydrogen phosphate ((NH_4_)_2_HPO_4_—Jin Shan Hua Shi—China) and dH_2_O as solvent [32]. Di-ammonium hydrogen phosphate and calcium nitrate tetrahydrate were dissolved in dH_2_O, obtaining P solution (3.58 M) and Ca solution (3.00 M), respectively. After 30 min, ammonium hydroxide (NH_3_, Sigma Aldrich, Saint Louis, MO, USA) was drop-wise added to adjust the medium alkalinity of P solution up to pH 9. To obtain a Ca/P ratio close to 1.67, P solution was added to Ca solution in specific proportions. CaP solutions were shaked (100 rpm, at 37 °C) until gelification. After that, the materials were dialyzed in 0.01 M phosphate buffered saline (PBS, Sigma Aldrich, Saint Louis, MO, USA) at pH 7.4 until equilibrated to the buffer pH and then lyophilized to obtain HAp. MaHA was dissolved at a concentration of 20 mg/mL in dH_2_O containing 0.1% wt/v of Irgacure 2959. Then, HAp were added to MaHA solution at different concentrations (1% and 2% wt/v) and the solutions were sonicated (Branson 1510 MT, Danbury, CT, USA) for 30 min to overcome the agglomeration of particles in the polymer. Composite SN disc-shaped samples were photocrosslinked for 180 s, rinsed in dH_2_O and stored at 4 °C.

Here in after, throughout this paper, neat MaHA and composite single networks will be labelled as SN and SNX, respectively where SN stands for single network and X represents the % wt/v of HAp, inside the network. As for example, SN1 stands for MaHA crosslinked network containing 1% wt/v of HAp. Similarly, neat and composite MaHA/PEGDA double networks will be labelled as DN and DNX, respectively, following the same sense. IPN hydrogels were also used as control samples for the mechanical study. They were prepared by one-step photocrosslinking for 60 s of a MaHA/PEGDA (1:10 wt:wt) water solution.

### 2.3. Preparation of Neat and Composite Double Network (DN) Hydrogels

Polymer and composite DN hydrogels were synthesized by two-step sequential network formation procedure as described by Gong et al. [25,26]. The polymer was dissolved in dH_2_O/Irgacure 2959 solution, at a concentration of 20 mg/mL. Disc-shaped samples were obtained by UV irradiating the MaHA hydrogels for 60 s. Separately, a PEGDA/dH_2_O solution (20% wt/v, Aladdin − M_w_ = 1 kDa), containing 0.1% wt/v of Irgacure 2959, was prepared. To obtain the DN blend, the SN disc-shaped hydrogels (1st network) were dipped in the PEGDA (2nd network) solution until reaching equilibrium for 4 days. The extracted discs were exposed to UV light for 300 s, washed in dH_2_O and stored at 4 °C. Similarly, composite hydrogels (containing 1% and 2% wt/v of HAp) were immersed in PEGDA solution and composite disc-shaped samples (DN1 and DN2) were prepared using the same technique by irradiating the system for 300 s.

### 2.4. Characterization of Crosslinked Hydrogels

#### 2.4.1. Thermogravimetric Analysis (TGA)

4–7 mg of the freeze-dried neat and composite materials was employed in order to perform TGA (TA Instruments Q 500, New Castle, DE, USA) The temperature was increased from 20 °C to 700 °C at a heating rate of 10 °C min^−1^ in a N_2_ atmosphere. From TGA results, the weight percentage of HAp in the SN hydrogel and the relative amount of MaHA and PEGDA in the DN were evaluated.

#### 2.4.2. Dynamic Mechanical Analysis (DMA)

TA-Q800 (TA-Instrument, New Castle, DE, USA) was employed to perform DMA. The frequency simulating the physiological stride frequency was set at 1.0 Hz. Furthermore, an amplitude of 50 μm in compression, a preload of 0.001 N and a force track of 120% were adopted. The tests were carried out in a closed chamber, in wet state, at room temperature. The fast testing procedure of only few minutes helps prevent sample dehydration. For each group, five disc-shaped samples (d = 8.0 ± 0.8 mm; h = 4.0 ± 0.5 mm) were obtained following the same procedure described above. The data were analyzed and expressed as mean value ± standard deviation.

#### 2.4.3. Scanning Electron Microscopy (SEM) 

SEM (S-800; Hitachi, Tokyo, Japan) was used to investigate neat, composite SN and DN hydrogels. Before analysis, the hydrogels were rinsed with MilliQ water, frozen by using liquid nitrogen, and lyophilized for 48 h. After that, an ultrathin sputter coating of Au/Pt was used. Energy dispersive X-ray spectroscopy (EDS) mapping was also employed to evaluate the distribution of HAp in the polymer matrix.

## 3. Results and Discussion

### 3.1. Synthesis of MaHA

**Figure 1:**
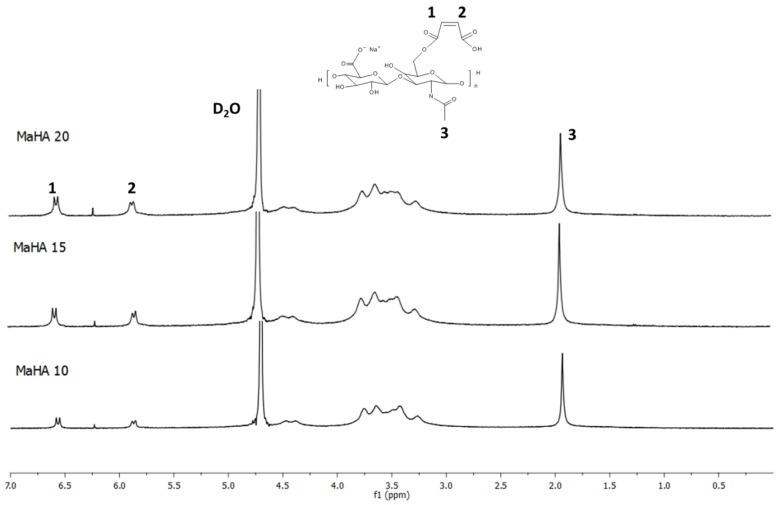
The ^1^H-NMR spectra of MaHA-based polymers with different degree of substitution (DS).

^1^H NMR spectra of MaHA with various DS are reported in Figure 1. The spectrum of HAs displays a characteristic peak at 1.9 ppm, which corresponds to the methyl protons (–CH_3_) of the N-acetyl group. This peak has been used as the reference to calculate the DS of modified polymers.

The peaks in the region from 3.3 ppm to 5.6 ppm correspond to the proton from D-glucuronic and N-acetyl glucosamine units. Looking at the spectra of MaHA with different DS, the two peaks at approximately 5.8 ppm and 6.5 ppm correspond to the vinylidene protons from MAA, confirming the successful attachment of the moiety on the HAs. Furthermore, it was possible to calculate the DS by comparing the area of the peaks belonging to MAA with the area of the –CH_3_ at 1.9 ppm. Results are reported in Table 1 and it is clear that by varying the amount of MAA used for reaction it is possible to tune the DS of the polymers. DS of 50.2%, 72.9% and 88.2% were achieved by using 10, 15 and 20 molar excess of MAA compared with the amount of primary –OH groups.

### 3.2. Characterization of Crosslinked Hydrogels

#### 3.2.1. Thermogravimetric Analysis (TGA)

**Figure 2:**
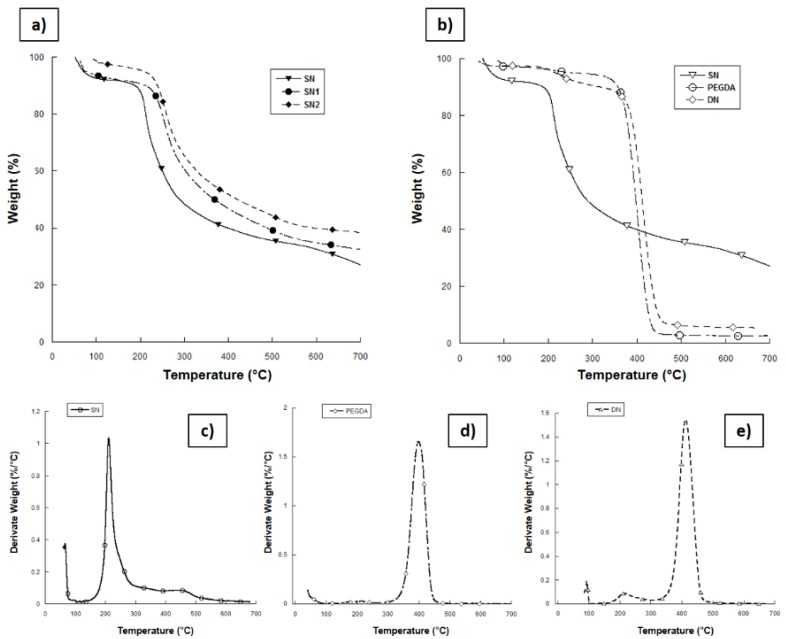
Results from thermogravimetric analysis (TGA) on lyophilized hydrogel samples. (**a**) Comparative curves for single network (SN) samples with different amounts of HAp; (**b**) comparative curves between SN, double network (DN) and poly(ethylene glycol) diacrylate (PEGDA) crosslinked materials; (**c**–**e**) derivative mass curves of SN, PEGDA, and DN hydrogels, respectively.

TGA of the composite networks was carried out at temperatures ranging from 20 °C to 700 °C under N_2_ (Figure 2a). The first weight loss, between 50 °C and 100 °C, can be ascribed to the change in water content, tightly bound to the chains of the highly hydrophilic biopolymers and trapped into the material even after lyophilization. Thermal decomposition of the MaHA matrix (SN) takes place at temperatures of approximately 200 °C and it is characterized by an intensive mass loss up to 300 °C. The maximum rate of mass loss corresponds to ~212 °C. The residual mass of 27.02 wt% found at 700 °C for SN network can be probably related to the production of coke residue during thermal decomposition [33]. As the ceramic phase is quite stable over 700 °C, the residual masses for SN1 and SN2 were respectively 32.03 wt% and 39.08 wt% and they can be used to determine the amount of HAp present in the networks.

By TGA, it is possible to assess the thermal degradation temperatures of the materials and also confirm the presence of different polymer components in the DN blend. Figure 2b shows the mass loss versus temperature curves for SN, PEGDA, and DN hydrogels. As previously described, mass loss near 100 °C is due to loss of the water from the hydrophilic polymer chain. SN samples show thermal degradation at around 225 °C (Figure 2c), while PEGDA network undergoes thermal degradation between 300 °C and 460 °C (Figure 2d). From TGA of the DN hydrogels (Figure 2b,e), it can be observed that the hydrogels show two weight losses: the first one between 150 °C and 300 °C is correlated with MaHA network degradation, while the second one between 300 °C and 460 °C is correlated with the degradation of PEGDA network. This confirms the absorption of PEGDA in the MaHA 1st network. Moreover, Figure 2e shows the derivate signal from TGA on DN blend, highlighting the great amount of PEGDA 2nd network inside the SN. It is worth noting that DN hydrogel showed higher thermal stability than SN materials probably due to the synergic crosslinking of both components.

#### 3.2.2. Dynamic Mechanical Analysis (DMA)

**Figure 3:**
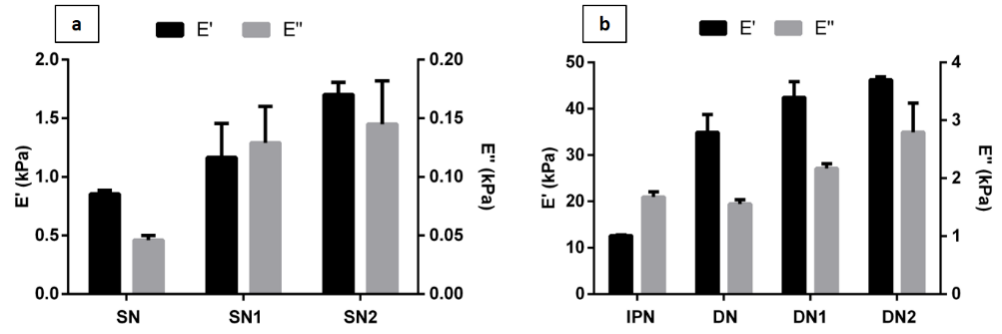
Results from DMA analysis. Storage modulus (E’) and loss modulus (E’’) of (**a**) neat and composite SN hydrogels with different hydroxyapatite nanoparticles (HAp) concentrations; (**b**) interpenetrated polymer network (IPN), neat and composite DN hydrogels with different HAp concentrations. Data are expressed as mean value ± standard deviation.

DMA was employed to compare mechanical properties of both DN and SN hydrogels as a function of material composition. Figure 3 reports the dynamic storage (E’) and loss (E’’) moduli of the hydrogels. All the hydrogel samples showed E’ higher than E’’, indicating an elastic solid behavior. The SN hydrogel is characterized by low compressive storage and loss moduli as 0.85 ± 0.03 kPa and 0.046 ± 0.004 kPa, respectively (Figure 3a). The storage modulus of the nanocomposite linearly increased with the HAp content, indicating that hydroxyapatite has a reinforcing effect on the elastic properties of neat hydrogel. For instance, in the case of the SN1 and SN2 nanocomposites, there was an increase of 36.5% and 98.2%, respectively compared to the neat hydrogel. The storage modulus spans from 0.85 ± 0.03 kPa for SN to 1.70 ± 0.10 kPa for SN2.

Figure 3b shows that the DN blend exhibited excellent overall mechanical properties. The DN storage and loss moduli are approximately 30-times higher compared to neat and composite SN hydrogels. This magnitude of mechanical enhancement is typical of DN hydrogels [24,25,34]. Incorporation of PEGDA in MaHA 1st network not only increased the density of polymer chains, but also increased the friction between chains, thus leading to higher storage moduli of DN hydrogels than SN ones [35]. Moreover, if compared to IPN network, DN showed an increase in elastic modulus by up a factor of 3 going from 12.6 ± 0.17 kPa to 34.9 ± 3.8 kPa (Figure 3b). Furthermore, the DN blend showed a slight increase in mechanical properties with the amount of HAp within the 1st. Indeed, the elastic modulus increased from 34.9 ± 3.8 kPa for DN to 46.3 ± 0.6 kPa for the DN2.

#### 3.2.3. Scanning Electron Microscopy (SEM)

**Figure 4:**
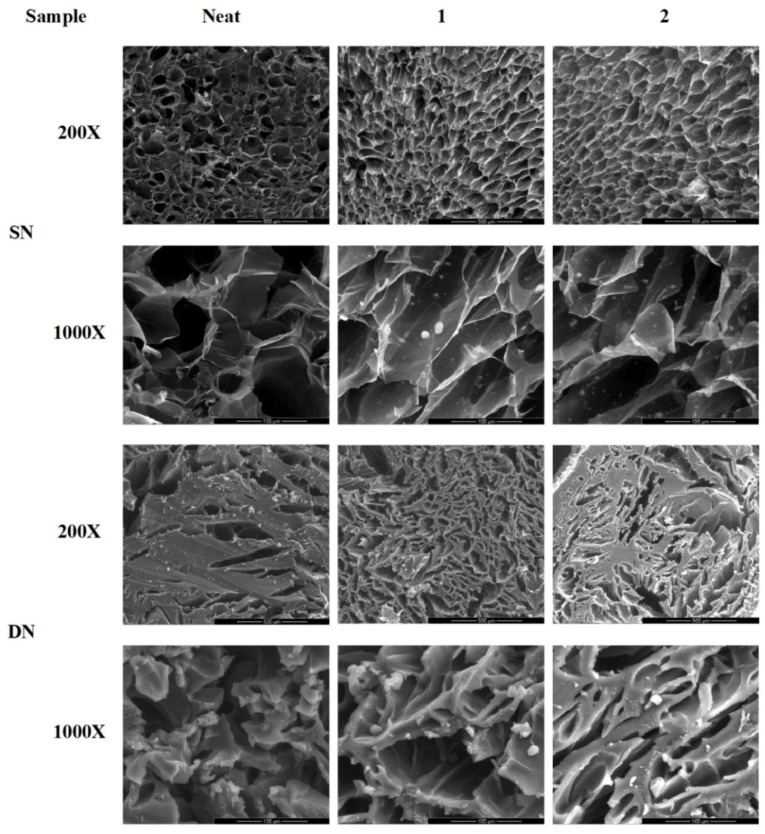
Representative scanning electron microscopy (SEM) images of: SN and DN hydrogels and related nanocomposites at different magnifications (200× and 1000×).

The morphology of dried hydrogels and HAp distribution were evaluated by SEM. Figure 4 shows representative SEM images of dried neat, composite SN and DN hydrogels in cross-section. The inner part of SN samples exhibited a three-dimensional (3D) open pore structure, while SN1 and SN2 samples showed the presence of HAp with relatively narrow size distribution. On the other hand, DN hydrogel showed a more compact 3D structure with thick pore walls and an average pore size ranging from 30 to 40 μm due to the presence of the second PEGDA network, which increased the relative crosslinking density of the structure (SEM images, magnification 1000×). Furthermore, SEM images showed that DN blends are less porous than SN networks. The increased density of the DN blends also explains the higher storage modulus if compared to SN hydrogels.

Moreover, SEM images of nanocomposite networks revealed the presence of HAp particles also on the surface of porous structure for both SN and DN hydrogels (Figure 4).

## 4. Conclusions

Here, HAs was chemically modified with photocrosslinkable motifs by reacting with maleic anhydride to obtain a maleated hyaluronic acid with improved mechanical properties.

The success of functionalization was highlighted by ^1^H NMR results, which showed the possibility to fine tune the reaction parameters to synthesize polymers characterized by different DS and improved stability due to the increased physical crosslinking points. However, to obtaining well-structured hydrogels with good mechanical performances, physical entrapment of inorganic particles (HAp) in the polymer network was performed.

To this aim, DN blends were suitably prepared by integrating a PEGDA into the previously synthesized MaHA hydrogel matrix, reinforced with HAp as inorganic filler. Due to the synergistic influence of DN structure and nanocomposite, the developed hydrogels are characterized by enhanced mechanical behaviour and thermal stability if compared to SN. The collective performance profile of the MaHA/PEGDA neat and composite DN hydrogels strongly suggests that this class of material has great potential as load-bearing materials for biomedical applications.

## Figures and Tables

**Table 1:** Reaction parameters and degree of substitution (DS) for maleated hyaluronic acid (MaHA).

Polymers	MAA/HAs (mol/mol)	DS (%)
MaHA 10	10/1	50.2
MaHA 15	15/1	72.9
MaHA 20	20/1	88.2
